# Comparison of Online-Onboard Adaptive Intensity-Modulated Radiation Therapy or Volumetric-Modulated Arc Radiotherapy With Image-Guided Radiotherapy for Patients With Gynecologic Tumors in Dependence on Fractionation and the Planning Target Volume Margin

**DOI:** 10.1001/jamanetworkopen.2023.4066

**Published:** 2023-03-22

**Authors:** Maja Guberina, Alina Santiago Garcia, Aymane Khouya, Christoph Pöttgen, Kostyantyn Holubyev, Toke Printz Ringbaek, Manfred Lachmuth, Yasemin Alberti, Christian Hoffmann, Julian Hlouschek, Thomas Gauler, Wolfgang Lübcke, Frank Indenkämpen, Martin Stuschke, Nika Guberina

**Affiliations:** 1 Department of Radiotherapy, West German Cancer Center, University of Duisburg-Essen, University Hospital Essen, Essen, Germany; 2 German Cancer Consortium, Partner Site University Hospital Essen, Essen, Germany

## Abstract

**Importance:**

Patients with newly diagnosed locally advanced cervical carcinomas or recurrences after surgery undergoing radiochemotherapy whose tumor is unsuited for a brachytherapy boost need high-dose percutaneous radiotherapy with small margins to compensate for clinical target volume deformations and set-up errors. Cone-beam computed tomography–based online adaptive radiotherapy (ART) has the potential to reduce planning target volume (PTV) margins below 5 mm for these tumors.

**Objective:**

To compare online ART technologies with image-guided radiotherapy (IGRT) for gynecologic tumors.

**Design, Setting, and Participants:**

This comparative effectiveness study comprised all 7 consecutive patients with gynecologic tumors who were treated with ART with artificial intelligence segmentation from January to May 2022 at the West German Cancer Center. All adapted treatment plans were reviewed for the new scenario of organs at risk and target volume. Dose distributions of adapted and scheduled plans optimized on the initial planning computed tomography scan were compared.

**Exposure:**

Online ART for gynecologic tumors.

**Main Outcomes and Measures:**

Target dose coverage with ART compared with IGRT for PTV margins of 5 mm or less in terms of the generalized equivalent uniform dose (gEUD) without increasing the gEUD for the organs at risk (bladder and rectum).

**Results:**

The first 10 treatment series among 7 patients (mean [SD] age, 65.7 [16.5] years) with gynecologic tumors from a prospective observational trial performed with ART were compared with IGRT. For a clinical PTV margin of 5 mm, IGRT was associated with a median gEUD decrease in the interfractional clinical target volume of −1.5% (90% CI, −31.8% to 2.9%) for all fractions in comparison with the planned dose distribution. Online ART was associated with a decrease of −0.02% (90% CI, −3.2% to 1.5%), which was less than the decrease with IGRT (*P* < .001). This was not associated with an increase in the gEUD for the bladder or rectum. For a PTV margin of 0 mm, the median gEUD deviation with IGRT was −13.1% (90% CI, −47.9% to 1.6%) compared with 0.1% (90% CI, −2.3% to 6.6%) with ART (*P* < .001). The benefit associated with ART was larger for a PTV margin of 0 mm than of 5 mm (*P* = .004) due to spreading of the cold spot at the clinical target volume margin from fraction to fraction with a median SD of 2.4 cm (90% CI, 1.9-3.4 cm) for all patients.

**Conclusions and Relevance:**

This study suggests that ART is associated with an improvement in the percentage deviation of gEUD for the interfractional clinical target volume compared with IGRT. As the gain of ART depends on fractionation and PTV margin, a strategy is proposed here to switch from IGRT to ART, if the delivered gEUD distribution becomes unfavorable in comparison with the expected distribution during the course of treatment.

## Key Points

**Question:**

What is the clinical benefit associated with online adaptive radiotherapy (ART) for gynecologic tumors compared with image-guided radiotherapy (IGRT)?

**Findings:**

This comparative effectiveness study comprising 7 consecutive patients with gynecologic tumors found that ART was associated with improved deviation of the delivered generalized equivalent uniform dose (gEUD) for interfractional clinical target volume values from the original dose distribution on the planning computed tomography scan compared with IGRT, without increasing the gEUD for the bladder and rectum for planning target volume margins less than 5 mm.

**Meaning:**

This study suggests that the advantage associated with ART depends on the number of applied dose fractions and planning target volume margins.

## Teaser

This comparative effectiveness study compares online adaptive radiotherapy technologies with image-guided radiotherapy for gynecologic tumors.

## Introduction

There is a need for high-precision, definitive percutaneous radiochemotherapy for patients with locally advanced cervical carcinomas or local recurrences of cervical carcinomas after surgery. In particular, the clinical target volume (CTV) of cervical and uterine cancer during external-beam radiotherapy may experience a high degree of deformations. Prospective trials of definitive radiochemotherapy for locally advanced cervical carcinomas or recurrences after surgery found that up to 20% or more of enrolled patients may have tumors that are unsuitable for a brachytherapy boost.^1,2,3,4^ Image-guided radiotherapy (IGRT) as a boost or as definitive treatment can be delivered either as conventionally fractionated or hypofractionated radiotherapy for these patients.^5,6,7,8,9^ However, higher-grade gastrointestinal toxic effects were found in some studies^5^ and only limited mature outcome data of this approach are currently available.^10^

The linear accelerator ETHOS (Varian Medical Systems) represents a new generation of radiotherapy linear accelerators. It comprises a new technique introducing artificial intelligence (AI)–enhanced adaptive replanning into clinical routine.^11^ Adaptive radiotherapy (ART) uses high-quality cone-beam computed tomography (CBCT), allowing precise daily IGRT and online adaptive replanning. In this way, it becomes possible to account for interfractional changes of tumor morphologic characteristics and healthy tissue deformations in the 3-dimensional space during online navigation on a daily basis.

To deliver definitive radiation doses above the equivalent dose in 2 Gy per fraction = 70 Gy (α/β = 10 Gy), steep dose gradients beyond the clinical tumor volume are necessary to minimize the high-dose volume in the bladder and rectal wall. Adaptive radiotherapy can adapt online to the anatomy of the day and markedly reduce internal target volume margins. At the same time, fast delivery of the dose after imaging must be assured to avoid intrafractional motion. Fast online adaptive methods are currently emerging. Commercial systems have become available to perform online magnetic resonance imaging–assisted or CBCT-assisted ART.^12,13^ To decrease adaptation time, automatic AI-based segmentation methods for organs at risk have been developed.^14,15^ However, data on the gain of ART in terms of the equivalent uniform dose and the accumulated dose distribution over the whole series in comparison with IGRT are lacking. Furthermore, strategies to optimally select patients based on dosimetric criteria for ART are also not defined. Last, the association of these systems with clinical outcomes and improvements in comparison with IGRT are largely unknown, to our knowledge.

The aim of the present study was to evaluate the feasibility of ART for treatment of patients with gynecologic tumors in the clinical setting. The recalculated dose distribution visualized on the daily CBCT scan, the structure-based dose deformations, and the value of these options for performing onboard navigation were evaluated in a patient cohort with gynecologic tumors. Target coverage and dose exposure of organs at risk were monitored in terms of the generalized equivalent uniform dose (gEUD).

## Methods

### Patient Cohort

This comparative effectiveness study is a nonrandomized controlled prospective observational study of the application of ART for gynecologic tumors with nonadaptive IGRT as the alternative dependent on the physician’s choice. It comprises all consecutive patients with gynecologic tumors who were treated with online ART from Janaury to May 2022 at the West German Cancer Center. After review by 2 expert radiation oncologists (M.G., C.P., or M.S.), ART was performed for clinical indications such as large recurrent or large residual tumor volumes in close proximity to critical organs at risk and the need for highly conformal protection of organs at risk during radiotherapy. All patients are part of the prospective, institutional clinical registry trial. Written informed consent was obtained from patients prior to the start of therapy. The study was conducted in accordance with the principles of the Declaration of Helsinki^19^ and was approved by the ethics committee of the University of Duisburg-Essen. This study followed the International Society for Pharmacoeconomics and Outcomes Research (ISPOR) reporting guideline.^16^ Treatment planning is described in detail in the eMethods in Supplement 1.

### Adaptive Radiotherapy

Treatment delivery on the ETHOS system can be performed in IGRT or ART mode.^11^ Relevant anatomical structures (so-called influencer organs) are automatically delineated based on an AI algorithm. All structures must be accepted by an expert radiation oncologist prior to plan approval and, if necessary, structures must be edited. Furthermore, daily CBCT scans serve for the online determination of interfractional CTV (iCTV) deformations compared with the planning CT scan. Based on the influencer organ structures, a deformation is created to adapt the CTV from the planning CT scan to the daily CBCT scan. The edits of propagated iCTVs from the planning CT scan to the CBCT geometry are classified as previously described^4^ on a 4-point scale from 0 to 3, as minor if modifications are required on few CT scan slices (<10%), intermediate if many slices require modifications, and major if many slices require larger edits or the structure needs a complete recontouring. The planning target volume (PTV) is created from the updated CTV as a 5-mm expansion. In the background, the system uses the CT-CBCT registration to deform the planning CT scan algorithm using velocity image deformation. A so-called synthetic CT scan is created by deforming the planning CT scan, which contains appropriate Hounsfield unit values for the photon dose calculation, to the anatomy of the day as imaged on the CBCT scan. On the synthetic CT scan a new plan is automatically optimized based on the clinical goals of the radiotherapy intent of the reference plan with special regard to the present expert-supervised, AI-segmented onboard anatomy of the day.^11,13^ For every fraction, the system delivers not only an adaptive-dose distribution but also a scheduled-dose distribution, which represents the dose from the reference plan recomputed on the new and possibly changed and deformed anatomy onboard. The adaptive-dose distribution differs from the scheduled-dose distribution by the reoptimization on the new anatomy.

Each fraction was assessed by the planning radiation oncologist specialist (M.G., C.P., or M.S.) or his or her deputy, from risk structures to target volume to plan geometry. At each session, the CTV and the PTV were adapted depending on the current anatomical setup and the position of the gross tumor volume by the planning specialist or his or her representative. In this way, plan variations could be optimally validated. In the plan selection step, the 2 generated plans, adapted and scheduled, are evaluated onboard and compared with the reference plan by the planning expert radiation oncologist and the planning medical physicist (M.G., A.S.G., C.P., K.H., M.L., W.L., F.I., or M.S.). After online, onboard plan selection and approval by the expert radiation oncologist and the medical physicist, a second CBCT scan is acquired and positioning once again verified to exclude intrafractional motion.

### End Points

The main end point was the added value of online ART in terms of dose coverage of the iCTV for fraction i, the dose that covers 95% (D95%), 98% (D98%), and 99% (D99%) of the interfractional PTV (iPTV) and iCTV and the volume that receives at least 95% of the prescribed dose (V95%) for the iCTV. Further end points are the assessment of dose and of the gEUD to organs at risk as a surrogate parameter for radiation-induced toxic effects. To account for acute toxic effects, all patients were monitored for therapy-induced tissue injuries during radiation treatment. Furthermore, toxic effects were reassessed 3 months after completion of therapy as part of routine follow-up. Acute toxic effects were evaluated with the European Organisation for Research and Treatment of Cancer–Radiation Therapy Oncology Group scoring system and long-term toxic effects were evaluated with the Fox Chase modification of the Radiation Therapy Oncology Group scoring system and the Late Effects Normal Tissue Task Force scoring system.

Gains of the adaptive vs the scheduled plans are given as Δ characteristic iCTV = % characteristic(A_i_)iCTV − %characteristic(S_i_)iCTV for dosimetric characteristics calculated from both the adaptive plan (A_i_) and the scheduled plan (S_i_) for the iCTV, where i indicates interfractional. The %characteristic(A_i_)iCTV or %characteristic(S_i_)iCTV is the percentage deviation of a dosimetric feature for the deformed iCTV at fraction i using the adaptive or scheduled plan, respectively, from that for the undeformed structure in the planning CT scan using the scheduled plan. Normalization is then performed by the dosimetric feature for the undeformed CTV in the planning CT scan. The concept of gEUD and normalization applied is more closely described in the eMethods in Supplement 1.

### Dose Accumulation

The treatment data for an adaptively implemented fraction, in which 1 or 2 CBCT scans were acquired, consist of the first and second CBCT scans, an ART plan and an IGRT plan, a rigid registration between the 2 CBCT scans, and a deformed registration from the last CBCT scan (secondary) to the planning CT scan (primary). The data presented in the monitoring module for each adaptive session were exported and analyzed with the software MIM Maestro, version 7.2.3 (MIM Software Inc). Dose accumulation was performed with the accumulation algorithm in the software MIM Maestro, version 7.2.3, with a resolution of 1 mm per voxel using the ETHOS-deformable registration of the daily CBCT scan with the target volumes of the day on the planning CT scan or, for comparison, a hybrid CTV contour–based and density-based deformation algorithm from MIM Maestro, version 7.2.3.

### Statistical Analysis

Descriptive and statistical analysis was performed using SAS, version 14.1 (SAS Institute Inc) and IBM SPSS Statistics, version 27 (IBM Corp). Given the nonnormal data distribution, the nonparametric Wilcoxon signed rank test for 2 dependent variables and the Mann-Whitney test for 2 independent variables comparing individual groups was used. A 2-sided *P* < .05 was considered statistically significant.

## Results

A total of 7 patients (mean [SD] age, 65.7 [16.5] years) with gynecologic tumors were treated over 10 treatment series with ART from January 1 to May 31, 2022. The Table summarizes classes of treatment indications for ART. All patients received at least 1 treatment series of at least 5 dose fractions using the adaptive mode. Two patients received more than 1 treatment series to reduce the treatment volume as sequential boosts. A total of 45 of 61 fractions of the adaptive series were delivered with the ART plan (73.8% applied adaptive fractions) and the other 16 fractions were delivered as IGRT. One patient was treated with the scheduled plan only, as anatomical deformations that made an adaptation necessary were negligible. The adaptive treatment took a mean (SD) of approximately 33 (5.4) minutes from opening the patient online file, CBCT scan acquisition, AI-based segmentation, plan generation and review, and second CBCT scan acquisition, to finalization of treatment delivery. Meanwhile, the iCTV structures required no editing in 41.0% (25 of 61) of propagated CTVs or minor editing in 36.1% (22 of 61) of propagated CTVs. A total of 18.0% (11 of 61) of propagated CTVs required intermediate editing and 3.3% (2 of 61) of propagated CTVs required major editing. Patients tolerated the online adaptive treatment well. The primary goals for coverage of the iCTV with the prescribed dose were V100% > 95% and D98% > 98%.

**Table.** Classes of Treatment Indications for ART

Indication for ART	No. (%)				Dose per fraction, Gy	Mean PTV (range), mL
	Patients (N = 7)	RT series (N = 10)	RT series with elective nodal irradiation (N = 10)	Fractions (N = 61)		
Recurrence of cervical or endometrial carcinoma not eligible for brachytherapy with infiltration of the rectal wall	2 (28.6)	2 (20.0)	0	10 (16.4)	2.0	204 (153-254)
Locally advanced cervical carcinoma with infiltration of the outer rectal wall, not eligible for brachytherapy	3 (42.8)	3 (30.0)	0	17 (27.9)	1.8	648 (375-850)
Locally advanced cervical carcinoma eligible for brachtytherapy or adjuvant RT of locally advanced cervical or endometrial carcinoma	2 (28.6)	5 (50.0)	3 (30.0)	34 (55.7)	1.8-2.0	1152 (232-2614)

Abbreviations: ART, adaptive radiotherapy; PTV, planning target volume; RT, radiotherapy.

**Figure 1.**
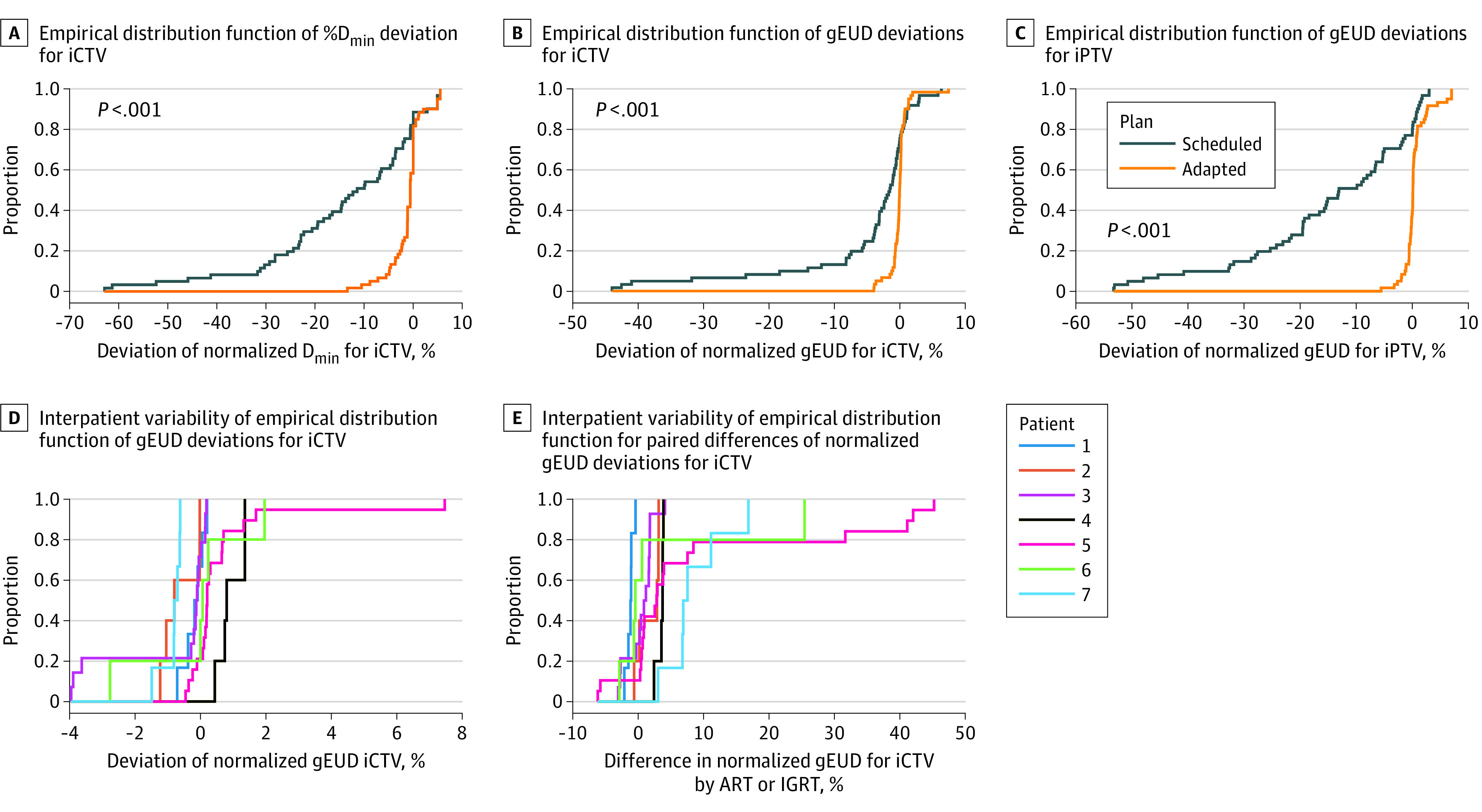
Empirical Distribution Functions A, Plot of empirical distribution function of percentage minimum dose deviation for interfractional clinical target volume (iCTV) for the adapted and scheduled plans (in comparison with the reference plan on the planning computed tomography [CT] scan). The empirical distributions differed significantly (*P* < .001; Kruskal-Wallis test). B, Plot of empirical distribution function of generalized equivalent uniform dose (gEUD) deviations for iCTV using the scheduled or adapted plans. The gEUD deviation values were normalized by the gEUD for the CTV in the planning CT scan and calculated from the dose-volume histograms using an exponent a = −20. The empirical distributions differed significantly (*P* < .001; Kruskal-Wallis test). C, Plot of empirical distribution function of normalized gEUD deviations for interfractional planning target volume (iPTV) using the scheduled dose distribution by the scheduled plan or adapted plans. The empirical distributions differed significantly between the scheduled and adapted plans over the fractions (*P* < .001; Kruskal-Wallis test). D, Interpatient variability (patients 1-7) of empirical distribution function of normalized gEUD deviations for iCTV using the adapted plan. The normalized interfractional gEUD deviation values were distributed over a small range from −4% to 8% compared with the planned dose distribution on the planning CT scan. E, Interpatient variability of the empirical distribution functions for the paired differences of interfractional normalized gEUD deviation values for iCTV using the adaptive and the scheduled plans. There was a significant interpatient variability in the gains of adaptation (*P* < .001; Kruskal-Wallis test). ART indicates adaptive radiotherapy; IGRT, image-guided radiotherapy.

Figure 1A highlights the empirical distribution of percentage deviation of the minimum dose for the iCTV from the dose-volume histograms of the scheduled and adaptive plans. The median percentage deviation of the minimum dose for the iCTV over the different fractions was −11.4% (90% CI, −45.9% to 4.9%) for the scheduled plan and −0.6% (90% CI, −8.0% to 5.2%) for the adapted plan. Differences in location were observed between the distributions of the percentage deviation of minimum dose for iCTV for the scheduled and the adaptive plans. The pairwise difference percentage deviation of minimum dose for the (A_i_)iCTV – percentage deviation of minimum dose for the (S_i_)iCTV had a median of 7.4% (90% CI, −0.9% to 48.5%) and differed significantly from 0 (*P* < .001; paired signed rank test).

Figure 1B delineates the empirical distributions of the percentage deviation of the normalized gEUD for the iCTV values (%gEUDiCTV) values, which differed significantly between the scheduled and adaptive plans in location. For a clinical PTV margin of 5 mm, IGRT was associated with a median gEUD decrease in the iCTV of −1.5% (90% CI, −31.8% to 2.9%) over all fractions. Online ART was associated with a decrease of −0.02% (90% CI, −3.2% to 1.5%), which was less than the decrease with IGRT (*P* < .001). The pairwise difference percentage deviation of normalized gEUD for the (A_i_)iCTV – percentage deviation of normalized gEUD for the (S_i_)iCTV had a median of 1.7% (90% CI, −3.0% to 36.4%) and differed significantly (*P* < .001; paired signed rank test).

To estimate the variation of the delivered percentage deviation of normalized gEUD for the iCTV with a PTV margin of 0 mm, we analyzed the dose coverage of the iPTV by the adaptive and the scheduled plans. Figure 1C shows the empirical distribution functions of the delivered percentage deviation of normalized gEUD for the iPTV for the adaptive and scheduled plans. For a PTV margin of 0 mm, the median gEUD deviation by IGRT was −13.1% (90% CI, −47.9% to 1.6%) compared with 0.1% (90% CI, −2.3% to 6.6%) with ART (*P* < .001). Comparison of Figure 1B and Figure 1C shows that the adaptative plan is associated with similar interfractional percentage of gEUD deviation distributions for the iCTV and the iPTV. However, the percentage deviation of normalized gEUD values with the scheduled plan were associated with a significant decrease from the iCTV to the iPTV (median, 8.6%; 90% CI, –2.0% to 26.3%; *P* < .001; paired signed rank test). The benefit associated with adaptation was larger for the percentage deviation of normalized gEUD for iPTV than for the percentage deviation of normalized gEUD for iCTV. The median gain of adaptation in terms of the change in the delivered gEUD for iCTV values was 1.7% (90% CI, −3.0% to 36.4%; *P* < .001; paired signed rank test) and the median gain of the change in the delivered gEUD for iPTV values was 14.1% (90% CI, −1.8% to 48.2%; *P* < .001; paired signed rank test). This finding suggests that adaption is more effective in conditions where the scheduled plans deteriorate, particularly treatments with smaller PTV margins. Interpatient variability of the empirical distributions of %gEUDiCTV using the adapted plans showed small variations in location (Figure 1D). The same was true for the scheduled plans. Figure 1E shows the gains of adaption in terms of change in the delivered gEUD for iCTV values for the different patients with significant differences from patient to patient.

**Figure 2.**
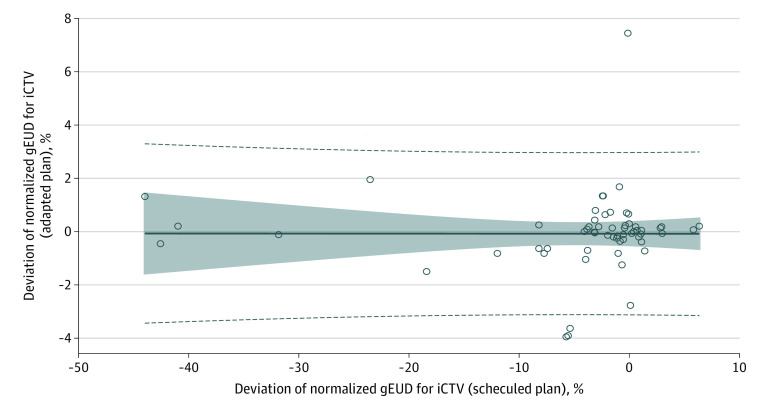
Scatterplot of Normalized Generalized Equivalent Uniform Dose (gEUD) Deviation Values for Interfractional Clinical Target Volume (iCTV) Using the Adapted Plan vs Normalized gEUD Deviation Values Using the Scheduled Plan The gEUD deviation values for iCTV using the adapted plan remained independent from those using the scheduled plan (*r* = 0.00). The dashed lines indicate 95% CIs for individual new estimates, the shaded area indicates the 95% CIs for expected estimated values, and the solid line indicates the adaptation line.

The percentage deviation of normalized gEUD for (A_i_)iCTV (%gEUDiCTV of the adaptive plans) remained independent from the decreases of the percentage deviation of normalized gEUD for (S_i_)iCTV (%gEUDiCTV of the scheduled plans) (Figure 2). The intercept and the slope of the linear regression curves were not different from zero and the correlation coefficient was *r* = 0.00.

The association between the percentage deviation of gEUD and the dosimetric parameters’ percentage deviation of the minimum dose, D99%, D98%, and V95% for the iCTV, which are associated with the cold spots within the iCTV, as well as the D95% for the iPTV, is delineated in eFigure 1A and B and Figure 2 in Supplement 1.

**Figure 3.**
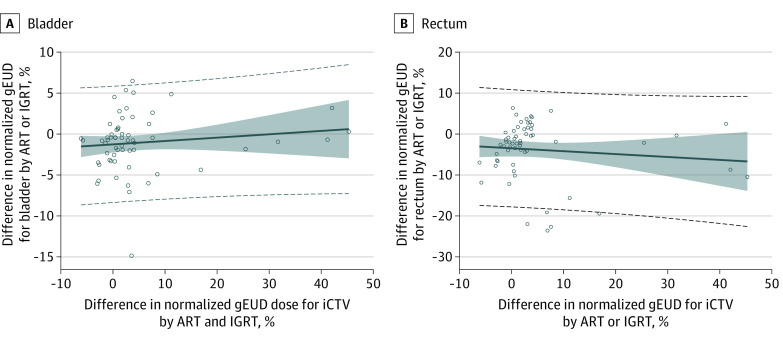
Change in Normalized Generalized Equivalent Uniform Dose (gEUD) for Interfractional Clinical Target Volume (iCTV) A, Scatterplot highlighting increase in the change in gEUD for iCTV using the adapted plan compared with the scheduled plan vs increase in change in the normalized gEUD for the bladder using the adapted plan compared with the scheduled plan. B, Increase in the change in gEUDiCTV using the adapted plan compared with the scheduled plan vs increase in change in the normalized gEUD for the rectum using the adapted plan compared with the scheduled plan. ART indicates adaptive radiotherapy; IGRT, image-guided radiotherapy. The dashed lines indicate 95% CIs for individual new estimates, the shaded area indicates the 95% CIs for expected estimated values, and the solid line indicates the adaptation line.

The association of normalized percentage deviation of gEUD with percentage deviation of D95% for the iPTV and with percentage deviation of the D98% values for the iCTV is shown in eFigure 3 and eFigure 4 in Supplement 1. The increase in the change in the gain of the gEUD for iCTV values with the adapted plan was not associated with an increase in the gEUD for the bladder or rectum (Figure 3A and B). On the contrary, the change in the gEUD values for the bladder was slightly lower by a median of −0.8% (90% CI, –6.2% to 4.9%) and for the rectum was slightly lower by a median of −2.2% (90% CI, –20.7% to 4.5%) with the adaptive plan in comparison with the scheduled plan (bladder, *P* = .009; rectum, *P* < .001; signed rank test). The correlation between change in the gEUD for iCTV values and change in the gEUD for the rectum as well as between change in the gEUD for iCTV and change in the gEUD for the bladder by ART was not significantly different from zero (rectum: *r* = −0.12, *P* = .47; bladder: *r* = −0.12, *P* = .34). eFigure 5 in Supplement 1 shows the variations of the position of minimum dose in the iCTV under the scheduled plans back-deformed to the CTV in the planning CT scan in z-axis and y-axis direction around the mean value per irradiation series.

**Figure 4.**
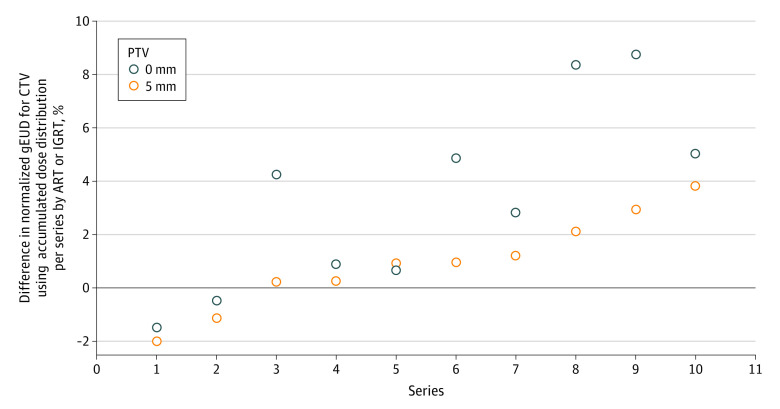
Change in Normalized Generalized Equivalent Uniform Dose (gEUD) for Clinical Target Volume (CTV) Values Comparing the Adaptive Plans With the Scheduled Plan Accumulated Over All Fractions per Series The change in gEUD for CTV values were obtained from the accumulated dose distributions for the adapted and scheduled plans for each of the 10 delivered treatment series and planning target volume (PTV) margins of 5 mm and 0 mm, respectively. The gain by adaption is larger for a PTV margin of 0 mm compared with 5 mm (*P* = .004; signed rank test for intraseries comparisons). ART indicates adaptive radiotherapy; IGRT, image-guided radiotherapy.

Next, we determined the gEUD values for the accumulated dose distributions over the adapted plans and the scheduled plans. For a PTV margin of 5 mm, the %gEUDCTV values for the accumulated dose distribution of the adapted plans ranged from −1.1% to 7.2% of the total prescribed dose, and for the scheduled plans ranged from −4.0% to 6.1% of the total prescribed dose, with no significant differences in pairwise comparisons (*P* = .13; signed rank test). However, for a PTV margin of 0 mm, the %gEUDCTV values for accumulated dose distributions of the adapted plans ranged from −3.7% to 6.2% of the total prescribed dose, and for the scheduled plans ranged from −7.9% to 1.2% of the total prescribed dose, with significant differences in pairwise comparisons (*P* = .02; signed rank test). Figure 4 shows the ΔgEUDCTV values comparing the adaptive plans with the scheduled plans using the accumulated dose distributions. The gain in gEUD for CTV by ART was plotted for the 10-treatment series of this study with PTV margins of 5 mm and 0 mm. The gain with adaption was larger for a PTV margin of 0 mm than for a PTV margin of 5 mm.

In a further step, we analyzed possible early stopping rules to switch from the IGRT using the scheduled plans to ART. Thus, we classified the %gEUDiCTV distribution from the scheduled plans with a PTV margin of 5 mm (Figure 1B) as acceptable for IGRT (distribution A), and the %gEUDiCTV distribution from the scheduled plans with a PTV margin of 0 mm (Figure 1C, distribution B) as inacceptable. For the scheduled plans, the percentage of %gEUDiCTV values less than −7% was 20% for distribution A and 59% for distribution B, but 0% for the distributions of the adaptive plans. Therefore, a possible strategy may be to switch from IGRT to ART if deviations in %gEUDiCTV values less than −7% occur a second time during the first 9 fractions or if such deviations occur in more than 20% of fractions thereafter. By this strategy, we would have switched to ART in 2 of the 10 series using a PTV margin of 5 mm and 4 of the 10 series using a PTV margin of 0 mm starting with the scheduled plan. According to this strategy, the %gEUDCTV for the accumulated dose distribution stayed above 95% in all series starting with scheduled plans and using PTV margins of 5 mm and 0 mm. Such strategies must be validated in the future with more data. They require delineation of the iCTV in the CBCT scans for dosimetric analyses, which also can be performed off-line between dose fractions.

Six patients have already received their first follow-up examination (4 of 7 patients who received the ART course and both patients who received the IGRT course). The median monitoring period of these patients was 114.8 days (range, 104-133 days), with no toxic effects reported.

## Discussion

Adaptive replanning is challenging in many ways, as it requires resources, time, and knowledge. The purpose of the present study was to examine the dosimetric gains of online ART using AI-based autosegmentation for treatment of patients with gynecologic cancer. Our results describe deviations between adaptive and scheduled plans from the reference plan in terms of gEUD depending on the PTV margin, with the adaptive plans being very close to the reference plan. The interfractional gEUD of the adaptive plans agreed excellently with the parameters of the reference plan. We demonstrated that the location of the minimum dose of the dose distribution in the CTV varies sufficiently from fraction to fraction, so that the worst-case dose accumulation with the minimum dose located for all fractions at the same position is prohibited. All accumulated dose distributions for series with PTV margins of 5 mm showed only small deviations, with %gEUDCTV values above −5%. A similar observation has been made for prostate cancer.^17^ However, with a PTV margin of 0 mm, deviations of the gEUD from the accumulated dose distribution occurred at a higher frequency of 40% of the treatment series, with the percentage deviation of gEUD values for CTV below −5%. The online ART mode is more important the smaller the PTV margin and the lower the number of delivered dose fractions.

Brachytherapy plays a central role in the curative treatment of cervical cancer.^18^ In EMBRACE-I (External Beam Radiochemotherapy and MRI Based Adaptive Brachytherapy in Locally Advanced Cervical Cancer), the median high-risk CTV was 28 cm^3^ (IQR, 20-40 cm^3^),^20^ compared with a more than 5-fold larger volume, depending on the patient, in the present study. However, in cases where large recurrent tumors need treatment, or where residual tumors remain after the first series or situations with extensive residual tumor infiltration of surrounding tissues, a brachytherapy boost is not applicable. We used ART for large residual tumors for which a brachytherapy boost was not applicable, or during the initial phase of pelvic radiotherapy including elective nodal regions.

The benefit associated with ART is greater for smaller PTV margins with higher sensitivities to uncertainties in the applied CTV deformations. The present study showed that when receiving hypofractionated radiotherapy with very small PTV margins, a certain percentage of patients with an individually worse %gEUDiCTV distribution than the overall population experienced benefits associated with online ART. Performing ART for each fraction of a series may be a strategy of low efficiency, particularly if IGRT with the scheduled plan results in equivalent good CTV dose coverage. Adaptive radiotherapy requires the availability of dedicated treatment machines; prolonged time slots per patient; the attendance of an interprofessional team of physicians, physicists, and technicians; and full attention during radiotherapy. Therefore, for the first time to our knowledge, we propose an offline strategy to monitor standard IGRT treatment and select patients who may benefit from online ART. The strategy is based on the observed dosimetric criteria using the scheduled plan during the initial dose fractions. In cases where %gEUDiCTV values are less than −7% in more than 20% of fractions throughout the series or in more than 1 fraction during the first 9 fractions or in over 20% of fractions thereafter throughout the whole series, IGRT should be switched to ART. With this strategy, the delivered gEUD for the CTV by the accumulated dose distribution remained above 95% in all series that started with IGRT and with PTV margins of 5 mm and 0 mm. This strategy prevented the delivered gEUD for the CTV by the accumulated dose distribution from decreasing below −5% and prevented unnecessary switching to ART in more than 80% of fractions using a PTV margin of 5 mm. Such a selection procedure does not require calculation of adaptive plans while the patient is being monitored. It requires adapting the iCTV to the daily CBCT scan and detecting %gEUDiCTV deviations less than a defined cutpoint of −7%, in this study, that can be performed remotely during offline review. At the same time, such a procedure would require less online-onboard supervision for every fraction by an experienced team of radiation oncologists and medical physicists than ART.

### Limitations and Strengths

This study has some limitations. A potential challenge for ART implementation in the clinical workflow is that the AI-based deformations need to be monitored by an experienced radiation oncologist to allow accurate assessment of CTV coverage. To date, automatic or semiautomatic AI-based monitoring cannot replace an experienced radiation oncologist and medical physicist. In addition, for successful implementation of the proposed strategy, it is important to calculate the interfractional gEUD after the monitored approval of iCTV values, each of which requires a thorough knowledge of the required subject matter and time.

This study also has some strengths. Our results are particularly important because they are, to our knowledge, the first real-world radiotherapy data on a linear-accelerator with VMAT adaptation, CT scan–based AI algorithms for online adaption implemented for patients with gynecologic cancer.

## Conclusions

This comparative effectiveness study suggests that ART based on daily CBCT scan improves %gEUDiCTV values per fraction compared with IGRT without increasing delivered gEUD values for surrounding healthy tissue in patients with gynecologic tumors. Greater benefits associated with ART compared with IGRT can be expected when PTV margins are less than 5 mm or fractionation schemes with fewer than 5 fractions are used. In addition, individual patients with a higher proportion of %gEUDCTV_i_ deviations below −7% should switch to ART. These patients can be identified early throughout the treatment course by using an offline %gEUDiCTV monitoring procedure in which %gEUDiCTV is estimated from daily CBCT images after each dose fraction.

## Notes

Supplement 1.
eMethods.

eReferences.
**eFigure 1A. **Linear-Quadratic Dependence of the %*g*EUDCTV_i_ on %D_min_ for the CTV_i _(%D_min_CTV_i_) **eFigure 1B. **Dependence of %*g*EUD on the %D99 for the CTV_i_ **eFigure 2. **Relation of the Increases in the Dosimetric Characteristics Indicated by Class (Δcharacteristic_class_CTV_i_) on the One and Increases in Δ*g*EUDCTV_i_ on the Other Hand Using the Adapted vs the Scheduled Plan for the Respective Dose Fraction: ΔD_min_CTV_i_ (Class 1), ΔD99CTV_i_ (Class 2), ΔD98CTV_i_ (Class 3), ΔD95PTV_i_ (Class 4) and ΔV95CTV_i_ (Class 5) **eFigure 3. **Dependence of the %*g*EUDCTV_i _ Values on the %D95 Values for the PTV_i_ (%D95PTV_i_) **eFigure 4. **Dependence of the %*g*EUD Values on the %D98 Values for the CTV_i _(%D98CTV_i_) **eFigure 5. **Deviations of the Position of D_min_ in z- and y-Direction Around the Mean Value per Irradiation Series

Supplement 2.
Data Sharing Statement
