# Supplementary material for: Comparison of Online-Onboard Adaptive Intensity-Modulated Radiation Therapy or Volumetric-Modulated Arc Radiotherapy With Image-Guided Radiotherapy for Patients With Gynecologic Tumors in Dependence on Fractionation and the Planning Target Volume Margin
Source: JAMA Netw Open. 2023 Mar 22;6(3):e234066. doi: 10.1001/jamanetworkopen.2023.4066 (PMC10034575; doi:10.1001/jamanetworkopen.2023.4066)
Supplement: Supplement 1. — eMethods. eReferences. eFigure 1A. Linear-Quadratic Dependence of the %gEUDCTVi on %Dmin for the CTVi (%DminCTVi) eFigure 1B. Dependence of %gEUD on the %D99 for the CTVi eFigure 2. Relation of the Increases in the Dosimetric Characteristics Indicated by Class (ΔcharacteristicclassCTVi) on the One and Increases in ΔgEUDCTVi on the Other Hand Using the Adapted vs the Scheduled Plan for the Respective Dose Fraction: ΔDminCTVi (Class 1), ΔD99CTVi (Class 2), ΔD98CTVi (Class 3), ΔD95PTVi (Class 4) and ΔV95CTVi (Class 5) eFigure 3. Dependence of the %gEUDCTVi Values on the %D95 Values for the PTVi (%D95PTVi) eFigure 4. Dependence of the %gEUD Values on the %D98 Values for the CTVi (%D98CTVi) eFigure 5. Deviations of the Position of Dmin in z- and y-Direction Around the Mean Value per Irradiation Series [file jamanetwopen-e234066-s001.pdf]

Supplementary Online Content

Guberina M, Santiago Garcia A, Khouya A, et al. Comparison of online-onboard adaptive intensity-modulated radiation therapy or volumetric-modulated arc radiotherapy with image-guided radiotherapy for patients with gynecologic tumors in dependence on fractionation and the planning target volume margin. *JAMA Netw Open.* 2023;6(3):e234066. doi:10.1001/jamanetworkopen.2023.4066

eMethods.
eReferences.
eFigure 1A. Linear-Quadratic dependence of the %*g*EUDCTV$_i$ on %D$_{min}$ for the CTV$_i$ (%D$_{min}$CTV$_i$)
eFigure 1B. Dependence of %*g*EUD on the %D99 for the CTV$_i$
eFigure 2. Relation of the increases in the dosimetric characteristics indicated by class (Δcharacteristic$_{class}$CTV$_i$) on the one and increases in ΔgEUDCTV$_i$ on the other hand using the adapted vs the scheduled plan for the respective dose fraction: ΔD$_{min}$CTV$_i$ (Class 1), ΔD99CTV$_i$ (Class 2), ΔD98CTV$_i$ (Class 3), ΔD95PTV$_i$ (Class 4) and ΔV95CTV$_i$ (Class 5)
eFigure 3. Dependence of the %*g*EUDCTV$_i$ values on the %D95 values for the PTV$_i$ (%D95PTV$_i$)
eFigure 4. Dependence of the %*g*EUD values on the %D98 values for the CTV$_i$ (%D98CTV$_i$)
eFigure 5. Deviations of the position of D$_{min}$ in z- and y-direction around the mean value per irradiation series

This supplementary material has been provided by the authors to give readers additional information about their work.

© 2023 Guberina M et al. *JAMA Network Open.*

eMethods.

Treatment planning

Patients with histopathologically proven gynecologic tumors were presented to the department of radiation therapy. After individual case discussion in the interdisciplinary panel and assessment by a radiation oncologist, patients were scheduled for radiotherapy. Patients underwent treatment planning simulation and received a planning-CT with or without contrast-agent, and advanced imaging techniques such as iterative-reconstruction and automatic-dose-modulation.

For the reproducible positioning special techniques such as constant bladder-fill and additional vaginal applicators were used. Treatment planning was performed using the ETHOS-treatment-planning-system (Varian, Palo Alto, US). GTV and CTV were defined and delineated according to the MR_{DG}-PreBT-MR/CT_{BT} environment following IBS-GEC ESTRO-ABS recommendations for CT-based contouring in image-guided adaptive brachytherapy for cervical cancer [1]. With this approach a clinical MR was available at initial diagnosis (1.5-3TMRI at diagnosis (MR_{DG})) and within few days prior to boost planning with the applicator in place (1.5-3T MRI at boost and brachytherapy planning (PreBT-MR or MR_{BT})). The diagnostic 1.5-3T MRI was conducted with a dedicated coil in supine position. T1-, T1 fat-saturated, contrast-enhanced and T2-weighted true-axial, sagittal, and coronal projections with 3-to 4mm thick slices, 0 to 1mm spacing, and depending on slice orientation 256*256 to 400*400 matrices. The MR-findings of residual tumor were taken side-by-side onto the high-resolution replanning-CT (CT_{BT}), also acquired few days prior to boost planning, to delineate the CTV-boost volume with respect to anatomical margins, where possible [1]. If residual tumors on MR_{BT} were too large for a brachytherapy boost or had residual infiltration of

© 2023 Guberina M et al. *JAMA Network Open.*

surrounding tissues incompatible for brachytherapy, patients underwent external beam therapy instead. The PTV was predefined as a 5-mm-expansion of the CTV to take potential set-up errors into account. Organs-at-risk were contoured on the planning- and replanning-CT to avoid dose hot spots inside the vulnerable regions. The automatic adaptive planning module generated volumetric-modulated arc radiotherapy (VMAT) and static-field intensity-modulated radiation therapy (IMRT) plans, following the clinical goals defined in the ETHOS RT-Intent. The ETHOS-system applies inverse planning with manual or templated application of optimization cost-function based objectives [2]. Dose was calculated with the Ethos AcurosXB (version 1.1.2.44, primary fluence-mode FFF, 6MV).

Special considerations of adaptive radiation therapy (ART) planning and treatment delivery

Treatment delivery on the ETHOS-system (Varian, Palo Alto, US) can be performed in image-guided radiotherapy (IGRT) or adaptive-mode [2]. While the former workflow allows for anatomic guidance based on the online patient position, the latter allows the onboard plan adaptation. Daily image guidance (IGRT) is an integral part of every radiation-therapy-sequence. Online target matching is achieved with the help of a 3-degrees-of-freedom-table. Rigid registration leads to an update of the isocenter position.

Equivalent-uniform-dose

We examined the EUD according to the phenomenological power-law-model (*g*EUD) [3] with tissue-specific parameters for the tumor and organs-at-risk (a = -20 for tumor; a = +7 for bladder and a = +7 for rectum) [4,5]. For a better inter-patient comparability $g\mathrm{EUD}_i$s were normalized to the prescribed dose. The percentage deviation of *g*EUD for

© 2023 Guberina M et al. *JAMA Network Open.*

the CTV_i (%gEUDCTV$_i$) and organs-at-risk at fraction i was determined for ART and IGRT in comparison to the reference-plan. The accumulated gEUD-values were examined over all fractions per patient. In this study, all adapted-plans, deformed images and structures were analyzed by an experienced team of medical physicists and expert radiation oncologists.

<u>Normalization</u>

All dosimetric characteristics for a deformed structure in the synthetic-CT (sCT) from dose fraction i of a series were given as the percentage deviation from the respective value for the undeformed structure in the planning-CT. All scheduled dose distributions were recalculated using the synthetic-CTs. Therefore, for example, the percentage deviation of D_{min} for the CTV_i at fraction i is

$$\%\mathrm{Dmin(Ai)CTVi} = \frac{100 * (\mathrm{Dmin(Ai)CTVi} - \mathrm{Dmin(S)CTV})}{\mathrm{Dmin(S)CTV}}$$

using the adaptive plan i. For the scheduled plan, the percentage deviation of D_{min} for the CTV_i at fraction i is

$$\%\mathrm{Dmin(Si)CTVi} = \frac{100 * (\mathrm{Dmin(Si)CTVi} - \mathrm{Dmin(S)CTV})}{\mathrm{Dmin(S)CTV}}$$

$D_{min}(S_i)CTV_i$ is the D_{min} in the deformed CTV (CTV_i) using the scheduled-plan recalculated on the synthetic-CT of fraction i. D_{min}(S)CTV is the D_{min} for the undeformed CTV in the planning-CT using the scheduled-plan. This normalisation was done, as dosimetric characteristics like D_{min}(S)CTV can show moderate deviations from the prescribed dose, varying from series-to-series. In addition, gains of the adaptive over the scheduled-plans were given as

© 2023 Guberina M et al. *JAMA Network Open.*

$$\Delta \text{characteristicCTVi} = \%\text{characteristic(Ai)CTVi} - \%\text{characteristic(Si)CTVi}$$

values for dosimetric characteristics calculated from both the adaptive (A_i) and the scheduled (S_i) plan for the CTV_i.

SUPPLEMENTARY RESULTS

In a next step, we analysed the relation between the %*g*EUD and the dosimetric parameters %D_{min}, %D99, %D98, and %V95 for the CTV_i, related to the cold-spots within the CTV_i as well as the %D95 for the PTV_i. Data points for the scheduled and adapted-plans follow the same relation with D_{min}, while the range of declines of the values was much smaller for the adapted than for the scheduled-plans (eFigure 1a). The coefficient of determination for the linear quadratic fit was 0.91. eFigure 1b shows the dependence of %*g*EUD on %D99 for the CTV_i. Only the linear term (p<0.0001, t-test) but not the intercept or the quadratic term became significant. The coefficient of determination R^2 for a linear quadratic fit was 0.90. The relation between %D99 and %*g*EUD-values for the CTV_i was close to a 1:1 relation with a slope of 1.193+/-0.038 (eFigure 1b).

eFigure 2 shows the linear quadratic relations between $\Delta D_{min}CTV_i$, $\Delta D99CTV_i$, $\Delta D98CTV_i$, $\Delta V95CTV_i$ and $\Delta D95PTV_i$ on the one and the ΔgEUDCTV_i-values on the other hand. All these percentage increases of the characteristics from the adaptive versus scheduled-plans over all fractions follow a monotonously increasing relation on ΔgEUDCTV_i. The largest coefficient of determination for a linear quadratic relation with ΔgEUDCTV_i was seen for $\Delta D99CTV_i$ (R^2=0.921), followed by $\Delta D98CTV_i$ (R^2=0.855), $\Delta D_{min}CTV_i$ (R^2=0.777), $\Delta V95CTV_i$ (R^2=0.760), and $\Delta D95PTV_i$ (R^2=0.569). From this

© 2023 Guberina M et al. *JAMA Network Open.*

and the close linear relation between $\%D99CTV_i$ and $\%gEUDCTV_i$, shown in eFigure 1b, it follows, that $\%D99CTV_i$ is a very favourable dosimetric parameter from the dose-volume histogram to describe the effectiveness of a dose fraction. In contrast to $\%D_{min}CTV_i$, $\%D99CTV_i$ is less dependent on the resolution of the dose matrix, the voxel size of the planning-CT and the CBCT–deformation-algorithm.

The relation of normalized $\%g$EUD on %D95 for the PTV_i and on %D98-values for the CTV_i is delineated in eFigure3 and eFigure4.

eFigure 5 shows the variations of the position of D_{min} in the CTV_i under the scheduled plans back deformed to the CTV in the planning-CT in z and y-direction around the mean value per irradiation series. As $\%D_{min}CTV_i$ is closely correlated with $\%gEUDCTV_i$, the positional variations of D_{min} are important for the result of dose accumulation. The more D_{min} randomly distributes over the CTV_i-margin from fraction-to-fraction, the less likely there is to be a worst-case accumulation of cold-spots where all D_{min} are at the same position. There were no differences in dispersions neither in y- nor in z-plane in dependence on the treatment series ($p>0.05$, Mood-test). The standard deviations are 22 mm in z-plane, 19 mm in y-plane and 24 mm in x-plane.

FURTHER DISCUSSION POINTS

Similar observations of dose fractionation to mitigate dosimetric variations within individual dose fractions have been reported for the interaction of time-dependent dose delivery and movements [6]. When both processes have random components, they affect the efficacy of a treatment series [6].

For patients with tumors unsuitable for brachytherapy there is a need of high precision external beam therapy to mimic brachytherapy [7-9]. For such dose escalated schedules the smallest possible PTV-margins below 5 mm must be aimed for. Online

© 2023 Guberina M et al. *JAMA Network Open.*

ART can realize here its full potential as shown in the present study. During ART image-quality must be sufficient for exact target-volume-delineation. Image-quality of the CBCT in this study was sufficient to delineate organs-at-risk and its anatomic relation with the residual tumor. Its boundaries, especially in lateral direction within the parametria and towards the pelvic wall, were precisely known from the planning MR_{DG}-PreBT-MR. In some cases, surgical clips helped to increase precision of CTV-delineation. If bladder or rectal wall were infiltrated, the infiltrated zones could be accurately detected on the high-quality CBCT. Our experience shows, that for online ART of gynecologic cancers a current MRI-study available for review of residual tumor on daily CBCT and on current diagnostic- CT is comparable with the precision of MRI-guided-brachytherapy. The same was found by others for image- guided- adaptive-brachytherapy [1, 10]. According to the IBS-GEC ESTRO-ABS-recommendations for CT-guided brachytherapy a diagnostic-MR prior to therapy, a pre-brachytherapy-MR within 1 week prior to brachytherapy and a CT with the applicator in place are comparable with the gold standard of MRI-guided approach for brachytherapy of cervical cancer [1]. Likewise, Mahantshetty et al. report that CT-based target and organ-at-risk delineation using MRI at diagnosis and real-time transrectal-ultrasound information during BT seems comparable with the gold standard MRI-guided-adaptive-brachytherapy for cervical cancer [11].

Only very limited data on ART is available at this time. Yock et al. used a software emulator to retrospectively analyze standard large pelvis radiotherapy for patients with cervical cancers using a PTV-margin of 5mm [12]. They found that D_{min} for the CTV of the primary and the nodal regions could be improved by adaptive radiotherapy by in average 0.25 ± 0.30 Gy per fraction, where the paired-differences of the scheduled with the adaptive-plan became significant [12]. Lakomy et al recently described their initial experience with online adaptive radiotherapy using an MR-linac in 10 patients with

© 2023 Guberina M et al. *JAMA Network Open.*

gynecologic cancer, treated with 90 fractions [13]. The treatment with online plan adaptation took in median 42 min. They used a PTV-margin of 3-5 mm and 17 of the fractions were treated with online-adapted plans. The mean accumulated dose of the GTV showed a decline above -5% in comparison to the reference plan [13].

Hadi et al. used a 0.35 Tesla MR-Linac for an online-adaptive boost with a PTV-margin of 5 mm for patients with gynecologic cancers ineligible for brachytherapy [14]. The median online treatment-time was with 77 min rather long. This treatment showed good tolerability. While these studies showed the feasibility of online adaptive radiotherapy, the on-coach times have to be shortened sufficiently to prevent intra-fraction organ shifts. In addition, though, MRI is considered the imaging modality of choice for gynecologic cancers, nowadays not 0.35 Tesla, but 1.5 and 3T-MRI is considered as minimum and optimum field strengths for sufficient diagnostic accuracy.

The median on-coach treatment-times in the present study with a CBCT-based adaptive treatment approach were 33 min using VMAT-optimization. Using a 12-field IGRT-optimization these times can be further shortened by approx. 8 min. The second CBCT for final verification, embedded within a MR_{DG}-PreBT-MR/CT_{BT} environment [1], combined with fast treatment delivery allows a nearly continuous intra-fraction monitoring.

eReferences.

1. Mahantshetty U, Poetter R, Beriwal S, Grover S, Lavanya G, Rai B et al. IBS-GEC ESTRO-ABS recommendations for CT based contouring in image guided adaptive brachytherapy for cervical cancer. Radiother Oncol. 2021;160:273-284.
2. Archambault Y, Boylan C, Bullock D, Morgas T, Peltola J, Ruokokoski E, et al. Making online Adaptive Radiotherapy Possible Using Artificial Intelligence and Machine Learning for Efficient Daily Re-Planning. Med Phys Int J 2020; 8: 77–86.
3. Niemierko A 1999 A generalized concept of Equivalent Uniform Dose (EUD) In: 41st Annual Meeting of the American Association of Physicists in Medicine, (Nashville, Tennessee aapm online)
4. Ghilezan M, Yan D, Liang J, Jaffray D, Wong J, Martinez A. Online image-guided intensity-modulated radiotherapy for prostate cancer: How much improvement can we expect? A theoretical assessment of clinical benefits and potential dose escalation by improving precision and accuracy of radiation delivery. Int J Radiat Oncol Biol Phys. 2004; 60:1602-10.
5. Arnaud A, Maingon P, Gauthier M et al. Image-guided IMRT for localized prostate cancer with daily repositioning: inferring the difference between planned dose and delivered dose distribution. Phys Med. 2014; 30:669-75.
6. Bortfeld T, Jokivarsi K, Goitein M, Kung J, Jiang SB. Effects of intra-fraction motion on IMRT dose delivery: statistical analysis and simulation. Phys Med Biol. 2002; 47:2203-20
7. Lőcsei Z, Sebestyén K, Sebestyén Z, Fehér E, Soltész D, Musch Z et al. IMAT-IGRT Treatment with Simultaneous Integrated Boost as Dose Escalation for Patients with Cervical Cancer: A Single Institution, Prospective Pilot Study. Pathol Oncol Res. 2021; 27:608446.
8. Albuquerque K, Tumati V, Lea J, Ahn C, Richardson D, Miller D et al. A Phase II Trial of Stereotactic Ablative Radiation Therapy as a Boost for Locally Advanced Cervical Cancer. Int J Radiat Oncol Biol Phys. 2020;106:464-471.
9. Morgenthaler J, Köhler C, Budach V, Sehouli J, Stromberger C, Besserer A, et al. Long-term results of robotic radiosurgery for non brachytherapy patients with cervical cancer. Strahlenther Onkol. 2021; 197:474-486.
10. Nesvacil N, Pötter R, Sturdza A, Hegazy N, Federico M, Kirisits C. Adaptive image guided brachytherapy for cervical cancer: a combined MRI-/CT-planning technique with MRI only at first fraction. Radiother Oncol. 2013; 107:75-81
11. Mahantshetty U, Poetter R, Beriwal S, Grover S, Lavanya G, Rai B et al. IBS-GEC ESTRO-ABS recommendations for CT based contouring in image guided adaptive brachytherapy for cervical cancer. Radiother Oncol. 2021;160:273-284.
12. Yock AD, Ahmed M, Ayala-Peacock D, Chakravarthy AB, Price M. Initial analysis of the dosimetric benefit and clinical resource cost of CBCT-based

© 2023 Guberina M et al. *JAMA Network Open.*

online adaptive radiotherapy for patients with cancers of the cervix or rectum. J Appl Clin Med Phys. 2021; 22:210-221.

13. Lakomy DS, Yang J, Vedam S, Wang J, Lee B, Sobremonte A et al. Clinical Implementation and Initial Experience With a 1.5 Tesla MR-Linac for MR-Guided Radiation Therapy for Gynecologic Cancer: An R-IDEAL Stage 1 and 2a First in Humans Feasibility Study of New Technology Implementation. Pract Radiat Oncol. 2022;12:e296-e305.
14. Hadi I, Eze C, Schönecker S, von Bestenbostel R, Rogowski P, Nierer L et al. MR-guided SBRT boost for patients with locally advanced or recurrent gynecological cancers ineligible for brachytherapy: feasibility and early clinical experience. Radiat Oncol. 2022; 17:8

© 2023 Guberina M et al. *JAMA Network Open.*

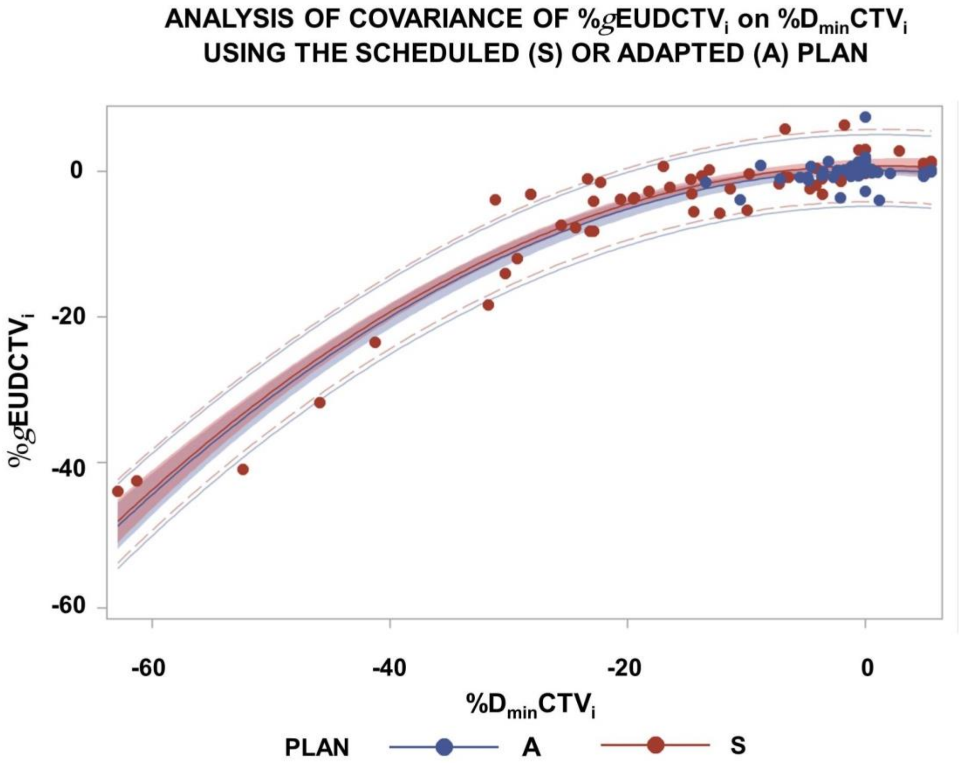

eFigure 1A. Linear-Quadratic Dependence of the %gEUDCTV$_i$ on %D$_{min}$ for the CTV$_i$ (%D$_{min}$CTV$_i$). The quadratic term was significantly smaller than 0 (p < 0.0001, t-test). Data points for the scheduled (red) and adapted plans (blue) follow the same relation, while the scatter of the %gEUD values of the adapted plans were much smaller than that of the scheduled plan.

© 2023 Guberina M et al. *JAMA Network Open.*

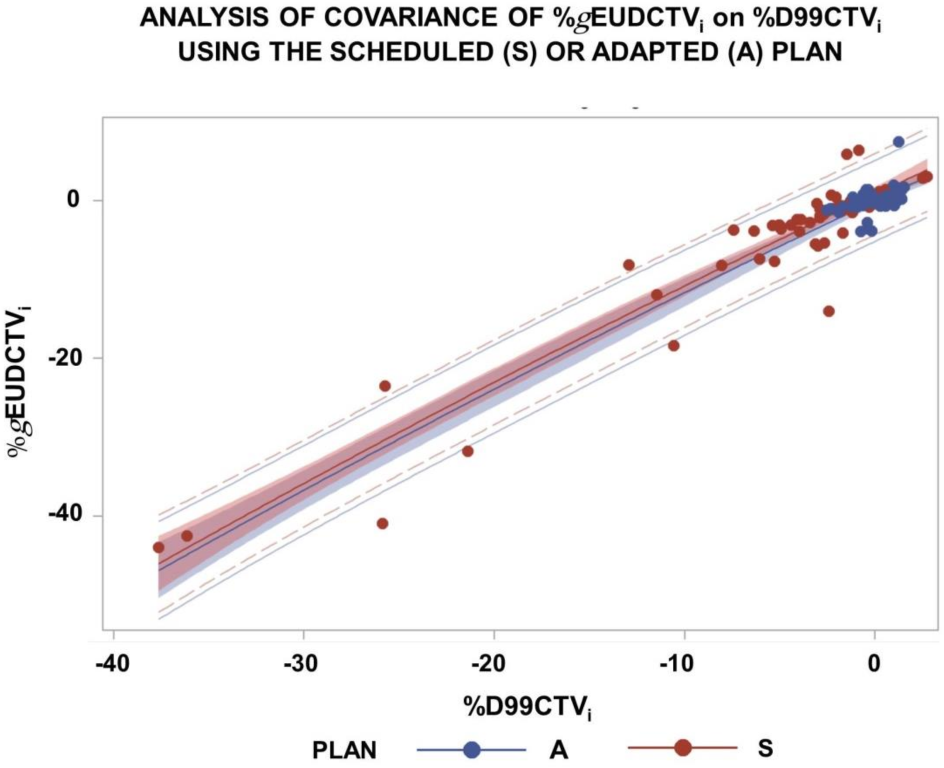

eFigure 1B. Dependence of %gEUD on the %D99 for the CTV_i. Only the linear term (*p* < 0.0001, t-test) but not the intercept or the quadratic term became significant. The relation was close to a 1:1 relation with a slope of 1.173 +/- 0.035.

© 2023 Guberina M et al. *JAMA Network Open*.

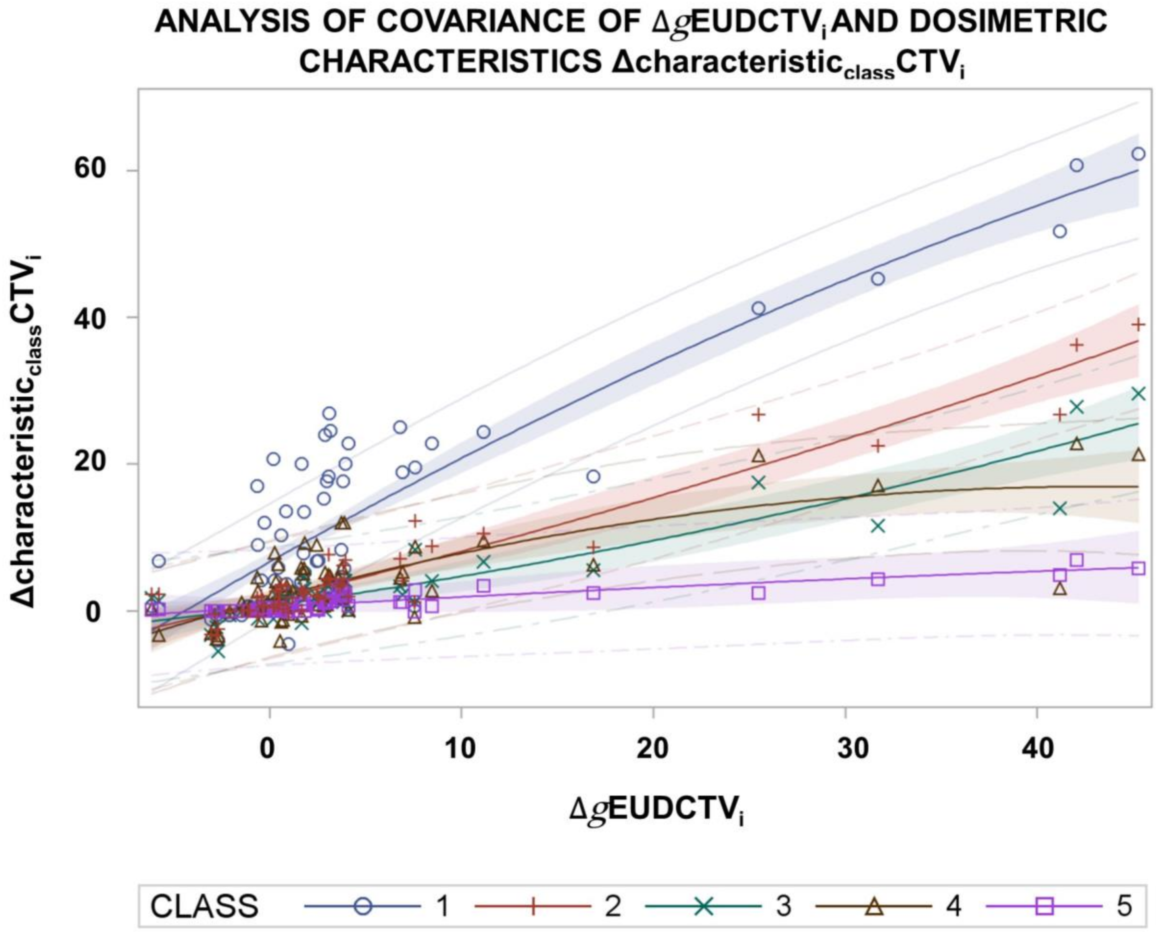

eFigure 2. Relation of the increases in the dosimetric characteristics indicated by class ($\Delta characteristic_{class}CTV_i$) on the one and increases in $\Delta gEUDCTV_i$ on the other hand using the adapted vs the scheduled plan for the respective dose fraction: $\Delta D_{min}CTV_i$ (Class 1), $\Delta D99CTV_i$ (Class 2), $\Delta D98CTV_i$ (Class 3), $\Delta D95PTV_i$ (Class 4) and $\Delta V95CTV_i$ (Class 5).

© 2023 Guberina M et al. *JAMA Network Open.*

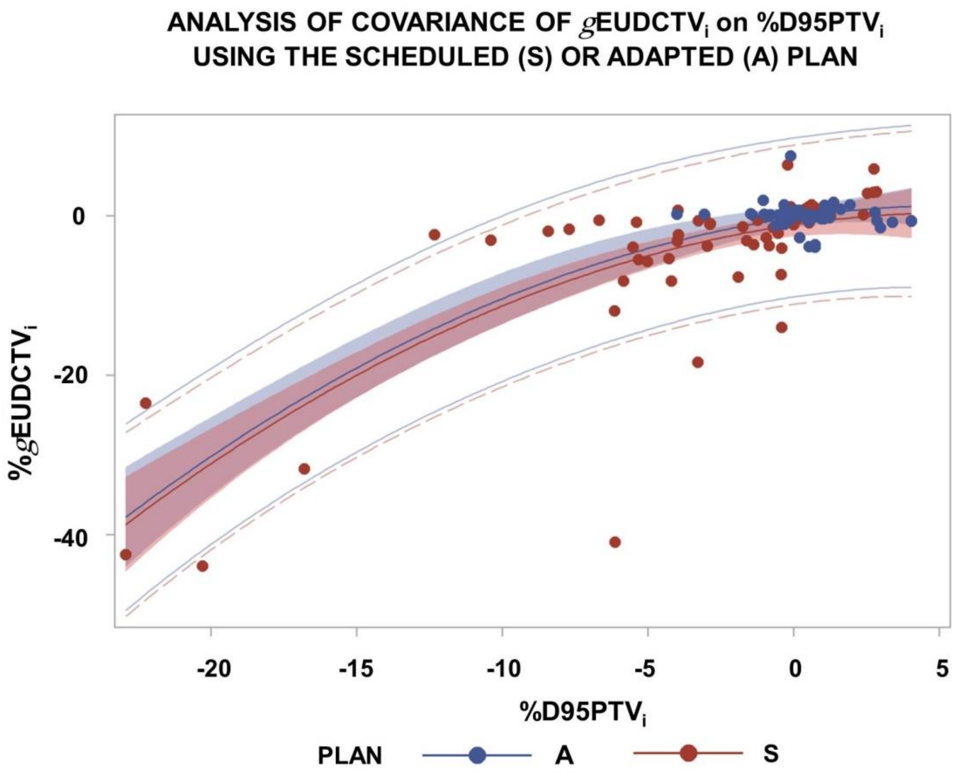

eFigure 3. Dependence of the %gEUDCTVi values on the %D95 values for the PTVi (%D95PTVi). Both, the linear 0 ($p < 0.022$, t-test) and the quadratic term 0 ($p = 0.0002$, t-test) were significantly different from zero. Data points for the scheduled (red) and adapted plans (blue) follow the same relation.

© 2023 Guberina M et al. *JAMA Network Open.*

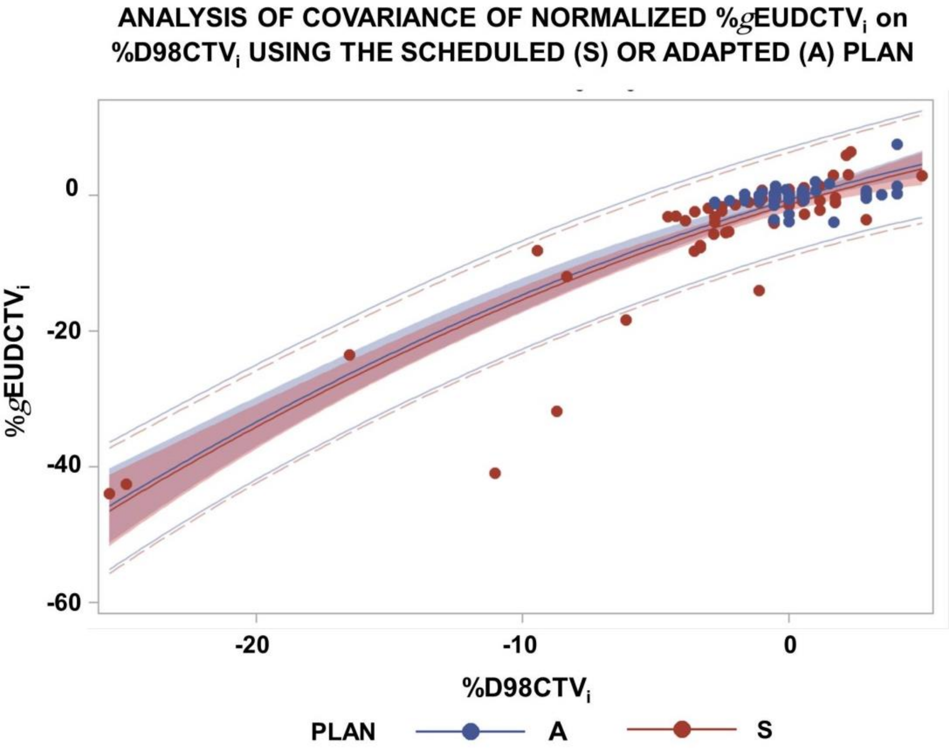

eFigure 4. Dependence of the %*g*EUD values on the %D98 values for the CTV$_i$ (%D98CTV$_i$). The linear ($p < 0.0001$, t-test) and the quadratic term 0 ($p = 0.003$, t-test) were significantly different from 0. Data points for the scheduled (red) and adapted (blue) plans follow the same relation.

© 2023 Guberina M et al. *JAMA Network Open.*

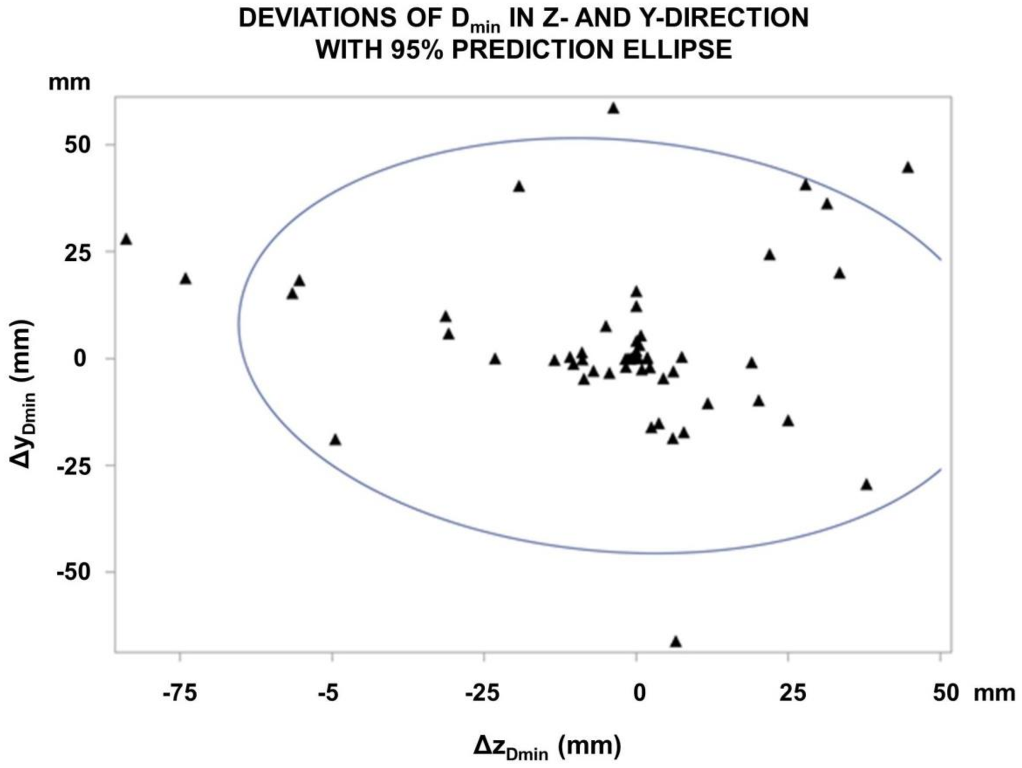

eFigure 5. Deviations of the position of D_{min} in z- and y-direction around the mean value per irradiation series. The deviations from the mean are plotted in y- and z-plane (scale in mm).

© 2023 Guberina M et al. *JAMA Network Open.*
