# Supplementary material for: Comparison of Online-Onboard Adaptive Intensity-Modulated Radiation Therapy or Volumetric-Modulated Arc Radiotherapy With Image-Guided Radiotherapy for Patients With Gynecologic Tumors in Dependence on Fractionation and the Planning Target Volume Margin
Source: JAMA Netw Open. 2023 Mar 22;6(3):e234066. doi: 10.1001/jamanetworkopen.2023.4066 (PMC10034575; doi:10.1001/jamanetworkopen.2023.4066)
Supplement: Supplement 2. — Data Sharing Statement [file jamanetwopen-e234066-s002.pdf]

Data Sharing Statement

Guberina. Comparison of Online-Onboard Adaptive Intensity-Modulated Radiation Therapy or Volumetric-Modulated Arc Radiotherapy With Image-Guided Radiotherapy for Patients With Gynecologic Tumors in Dependence on Fractionation and the Planning Target Volume Margin. *JAMA Netw Open*. Published March 22, 2023. doi:10.1001/jamanetworkopen.2023.4066

Data
Data available: Yes
Data types: Data dictionary
How to access data: nika.guberina@uk-essen.de
When available: With publication

Supporting Documents
Document types: Other (please specify)
Additional Information: primary data analysis evaluation matrix and mask
How to access documents: nika.guberina@uk-essen.de
When available: With publication

Additional Information
Who can access the data: researchers whose proposed use of the data has been approved
Types of analyses: specific purposes dedicated to improvement in RT of gynecological tumors
Mechanisms of data availability: after approval of a proposal
